# Do HIV-specific CTL continue to have an antiviral function during antiretroviral therapy? If not, why not, and what can be done about it?

**DOI:** 10.3389/fimmu.2013.00052

**Published:** 2013-03-01

**Authors:** Dorian McIlroy

**Affiliations:** EA4271 Laboratoire d’Immunovirologie et Polymorphisme Génétique, Faculté de Médecine et de Pharmacie, Université de Nantes, LUNAM UniversitéNantes, France

**Keywords:** HIV, CTL antiretroviral therapy

## Abstract

Pharmacological reactivation of human immunodeficiency virus (HIV) expression from latent proviruses coupled with fully suppressive antiretroviral therapy (ART) has been suggested as a strategy to eradicate HIV infection. In order for this strategy to be effective, latently infected cells must be killed either by the cytopathic effect of reactivated HIV gene expression, or by HIV-specific cytotoxic T lymphocyte (CTL). However, a review of current data reveals little evidence that CTL retain an antiviral effector capacity in patients on fully suppressive ART, implying that the HIV-specific CTL present in these patients will not be able to eliminate HIV-infected CD4^+^ T cells effectively. If this is due to functional impairment or a quantitative deficit of HIV-specific CTL during ART, then therapeutic vaccination may improve the prospects for eradicating latent reservoirs. However, data from the macaque simian immunodeficiency virus (SIV) model indicate that *in vivo*, SIV-specific CTL are only effective during the early stages of the viral replication cycle, and this constitutes an alternative explanation why HIV-specific CTL do not appear to have an impact on HIV reservoirs during ART. In that case, immunotoxins that target HIV-expressing cells may be a more promising approach for HIV eradication.

## INTRODUCTION – ANTIRETROVIRAL THERAPY, ERADICATION STRATEGIES, AND THE POSSIBLE ROLE OF HIV-SPECIFIC CTL

Current regimens of antiretroviral therapy (ART) are able to suppress human immunodeficiency virus (HIV) replication to levels that are practically undetectable, and many observations indicate that complete inhibition of active HIV replication is indeed attained in patients on prolonged ART (McMahon et al., 2010; Yukl et al., 2010; Hatano et al., 2011; Besson et al., 2012; Gandhi et al., 2012).

Unfortunately, the integration of replication-competent HIV proviral genomes into host cell DNA creates a viral reservoir that is unaffected by ART (Chun et al., 1997, 2005; Finzi et al., 1997; Viard et al., 2004). These proviruses can be reactivated when the host T cell is stimulated by antigen or by cytokines (Chun et al., 1998; Wang et al., 2005), and as this inevitably occurs in a normal environment, HIV replication continually reignites from the smoldering ember of proviral DNA, leading to rebound viremia and a resumption of disease progression when ART is discontinued. This implies that eradication of the proviral reservoir will be a necessary part of therapeutic strategies aiming to cure HIV infection (Trono et al., 2010; Deeks et al., 2012). To this end, several groups have demonstrated that histone deacetylase (HDAC) inhibitors (Lehrman et al., 2005; Contreras et al., 2009), protein kinase C agonists (Kulkosky et al., 2001; Korin et al., 2002), and cytokines that activate the Janus kinase/signal transducers and activators of transcription (JAK/STAT) signaling pathway (Scripture-Adams et al., 2002; Wang et al., 2005) can reactivate HIV expression from latent proviral genomes. The activity of one HDAC inhibitor, Vorinostat (also known as suberoylanilide hydroxamic acid, or SAHA), has been demonstrated in patients (Archin et al., 2012), strongly indicating that this may be a clinically viable approach.

However, this strategy for the eradication of HIV hinges on the assumption that latently infected cells will be killed by the reactivation of latent proviruses, either as a result of cytopathic effects of HIV gene expression, or through lysis by HIV-specific cytotoxic T lymphocyte (CTL). In a recent landmark study, Siliciano and colleagues found that reactivation of HIV provirus by Vorinostat was not cytopathic, and that elimination of latently infected cells required their recognition and lysis by functionally competent HIV-specific CTL (Shan et al., 2012). The success of clinical trials aiming to eradicate latent reservoirs of HIV infection may therefore depend on the presence of functional HIV-specific CTL in patients who have experienced several years of ART.

Four distinct lines of evidence indicate that CTL limit HIV replication in both the acute and chronic phase of untreated HIV infection. These are: (1) associations between human leukocyte antigen (HLA) class I alleles and disease progression; (2) HIV sequence evolution and polymorphism indicating selection pressure exerted by CTL *in vivo*; (3) inverse correlation of viral load (VL) with Gag-specific CTL responses; and (4) ability of CTL to inhibit HIV replication *ex vivo*.

For each of these types of evidence, I will briefly summarize what the approach has revealed about the CTL response during untreated HIV infection, then review the relevant data in patients on ART, in order to assess to what extent HIV-specific CTL continue to have an antiviral function during ART. In the light of this data, two alternative strategies aiming to eradicate infected cells with reactivated latent infection will be discussed.

## ASSOCIATIONS BETWEEN HLA CLASS I ALLELES AND DISEASE PROGRESSION

In untreated HIV infection, associations between HLA genetic polymorphism and disease progression were identified in several studies, using both candidate gene (reviewed in Carrington and O’Brien, 2003), and genome-wide strategies (The International HIV Controllers Study, 2010). The observation that HLA class I genotype has a strong influence on the progression of HIV infection suggested that CTL play an important role in the natural history of the disease. The HLA-B polymorphisms associated with disease progression influence the peptide repertoire presented by the allele, indicating that favorable HLA-B alleles, such as B27, B57, and the wider Bw4 allele group, confer protection to HIV disease because they present the “best” HIV epitopes to CTL – that is, those epitopes in which escape mutations incur a high fitness cost to the virus (Carrington and Walker, 2012; Goulder and Walker, 2012).

Although ART effectively halts clinical disease progression in the majority of patients, its success rate is not 100%, and a significant proportion of patients on ART experience either immunological failure, defined as poor or no recovery of CD4^+^ T lymphocyte count, or virological failure, characterized by the persistence of plasma virus. If CTL lysis of infected cells contributes to the efficacy of treatment, then one should expect genetic associations between HLA class I alleles and treatment outcome. Four published studies have tested this hypothesis (Brumme et al., 2007; Ahuja et al., 2008; Rauch et al., 2008; Kuniholm et al., 2011), and the results are summarized in **Table 1**. Overall, none of the HLA alleles associated with protective effects in untreated HIV infection are associated with positive treatment outcomes in patients on ART. Indeed, the associations that have been most consistently reported are between the presence of “protective” HLA-B alleles and higher risk of immunological failure in patients on ART. In contrast, the cysteine–cysteine chemokine receptor 5 (CCR5Δ32) polymorphism was associated with better virological response to ART (Brumme et al., 2005; Laurichesse et al., 2007).

**Table 1:** Reported associations between HLA alleles and immunological and virological response to ART.

Cohort size	Immunological response	Virological response	Reference
765	Slower CD4^+^ recovery in patients homozygous at any HLA class I locus	No association	Brumme et al. (2007)
502	Slower CD4^+^ recovery in patients carrying HLA-B57	Not tested	Ahuja et al. (2008)
265	Slower CD4^+^ recovery in patients homozygous for the Bw4 allele group	No association	Rauch et al. (2008)
860	Slower CD4^+^ recovery in patients homozygous for the Bw4 allele group Tendency toward poorer immunological response in patients carrying B5701 and B5801	Patients carrying B5701 and B5801 less likely to attain VL < 500 copies/mL within 12 months of treatment initiation	Kuniholm et al. (2011)

The physiological interpretation of these results is far from clear. It is intriguing that in two studies (Ahuja et al., 2008; Kuniholm et al., 2011), the same alleles that are associated with better control of viral replication and maintenance of CD4^+^ T cell counts in untreated HIV infection were associated with poorer CD4^+^ T cell recovery during therapy. One scenario that could explain these observations is that HIV-specific CTL continue to lyse cells presenting their cognate HLA–peptide complex during ART, but that this is detrimental, rather than beneficial, for CD4^+^ T cell recovery, so that the more efficient the anti-HIV CTL response, the more CD4^+^ T cell recovery is impaired.

## HIV SEQUENCE EVOLUTION INDICATING SELECTION PRESSURE FROM CTL

Studies of HIV sequence evolution over time (Borrow et al., 1997; Goulder et al., 1997; Cao et al., 2003; Allen et al., 2005), and associations between HIV sequence polymorphisms and the HLA type of the infected person (Moore et al., 2002; Brumme et al., 2009), have shown that escape from CTL responses occurs during HIV infection by the selection of mutations in and around CTL epitopes. These mutations either abrogate peptide binding to the presenting HLA, influence processing of the epitope, or prevent binding of the HLA–peptide complex to its cognate T cell receptor (TCR; Goulder and Watkins, 2004), and their emergence indicates that CTL exert selection pressure on the viral quasispecies *in vivo*. Multiple HLA-A and HLA-B alleles are associated with escape mutations (Moore et al., 2002; Brumme et al., 2009), showing that CTL restricted by non-protective HLA alleles do in fact have an impact on HIV replication in infected patients. Fitness constraints limit the number of possible escape variants that are available to the virus, and the protective alleles HLA-B27, 5701, and 5801 target epitopes with few viable escape variants (Brockman et al., 2007; Miura et al., 2009), which to a large extent explains the associations of these alleles with slower disease progression.

Two studies found statistical associations between patient’s HLA, and mutations that accumulated in either the protease or the reverse transcriptase (RT) proteins during virological failure (John et al., 2005; Mueller et al., 2007), indicating that the CTL response influences the emergence of drug resistance mutations. More recently, a powerful longitudinal analysis of virus sequences from 619 patients, sampled before and during ART was reported (Knapp et al., 2012). HIV protease and RT sequences were obtained from plasma samples with VL > 1000 copies/mL, and a median of five post-ART samples were sequenced per patient. The availability of pre-treatment sequences made it possible to determine unequivocally whether CTL escape mutations had been selected before or during ART. Overall, 43% of patients developed at least one additional CTL escape mutation over the period on ART (median 5.1 years), and the mean rate of accumulation of CTL escape mutations was estimated to be sevenfold slower during ART, compared to before treatment initiation.

In the context of successful ART, the low levels of plasma VL pose a technical challenge for longitudinal analysis of HIV RNA sequences. Two published studies did however succeed in just such an undertaking, using ultracentrifugation to concentrate virus particles from plasma before RNA extraction. Casazza et al. (2005) studied the sequences of six CTL epitopes in three patients with VL < 400 copies/mL, and found evidence of sequence evolution in CTL epitopes during ART in one of three patients studied. Similarly, in a more recent study of 20 patients with VL < 50 copies/mL, sequences were obtained for 12 study participants, and evidence for selection of CTL escape mutations was found in three patients (Shiu et al., 2009).

These five studies all found evidence of accumulation of CTL escape mutations during ART, with the rate of accumulation correlated with the level of ongoing viral replication in two studies (Shiu et al., 2009; Knapp et al., 2012). This observation may indicate that at lower VLs, CTL responses exert less pressure on the virus, due to the reduced number of circulating HIV-specific CTL (see below). On the other hand, as virus replication is suppressed, fewer mutations arise, and escape mutations accumulate more slowly. As an extreme example, in patients with complete suppression of viral replication, no escape mutations can arise because the virus no longer has the means to respond to any type of selection pressure – but that does not imply that the selection pressure has ceased to exist.

The pressure that CTL exert on HIV therefore appears to be maintained during ART and can be detected in some patients even when VL is reduced to <50 copies/mL. However, the relative potency of this selection pressure (as strong as, or weaker than that observed during primary or chronic infection) cannot be inferred from these studies.

## ASSOCIATIONS BETWEEN PRESENCE OF CTL AND PLASMA VL

Cytotoxic T lymphocyte can exert selection pressure on the virus only if they are in fact present in the infected person, and evidence for the antiviral effect of CTL in untreated HIV infection was also provided by documenting associations between the presence of HIV-specific CTL and lower VL. During acute infection, the appearance of HIV-specific CTL coincides with the reduction of VL from its peak to its set-point value (Borrow et al., 1994; Koup et al., 1994), and in chronic infection, cross-sectional studies in many different cohorts showed that the number of Gag-specific CTL is inversely correlated with VL (Klein et al., 1995; Ogg et al., 1998; Buseyne et al., 2002; Edwards et al., 2002; Zuñiga et al., 2006; Kiepiela et al., 2007).

Following the initiation of ART, the numbers of circulating HIV-specific CTL decline with two-stage kinetics (Ogg et al., 1999; Casazza et al., 2001), so that the number of circulating HIV-specific CTL is reduced 5- to 10-fold over the first 1–2 years of ART. This indicates that antigenic stimulation maintains the levels of CTL observed in untreated patients, so that when virus replication is suppressed by ART, CTL responses wane. However, HIV-specific CD8^+^ T lymphocytes do not entirely disappear in patients on ART, and HIV-specific CTL can be detected in many patients after several years of effective ART (Appay et al., 2002; Oxenius et al., 2002; Rehr et al., 2008). Two studies have addressed whether these “residual” HIV-specific CTL responses might be related to residual virus replication during suppressive ART. Both found that residual viral replication during ART, measured either by the amount of intracellular HIV RNA (Patterson et al., 2001), or the occurrence of transient low-level (50–1000 copies/mL) viremic blips (Papasavvas et al., 2006), was not related to the presence of CD8^+^ T cell responses.

In the context of partially effective ART, the available data are somewhat equivocal. One study found that in patients with low VL on ART (200 < VL < 10,000 copies/mL), or virological failure (VL > 10,000 copies/mL), Gag-specific CD8^+^ T cells were inversely correlated with VL (Alatrakchi et al., 2005), and overall, Gag-specific CD8^+^ responses were stronger in patients with partial control of viral replication compared to those with full virological failure. Similar results were found in a study of pediatric patients (Buseyne et al., 2005), whereas a third study found that the Gag-specific interferon-gamma (IFN-γ) response in CD8^+^ T cells was not different in partial controllers (VL 500–6000 copies/mL) compared to patients with uncontrolled HIV replication (>10,000 copies/mL; Emu et al., 2005). In all three studies, however, Gag-specific CD8^+^ T cells responses were lower in patients with VL < 200 copies/mL, indicating that a certain threshold of persistent HIV replication is required to reactivate HIV-specific CTL during ART (Jin et al., 2000).

Although the number of studies addressing this issue is small, none of them found an association between the presence of HIV-specific CTL, and the absence of residual viral replication during suppressive ART. This implies that any effect of HIV-specific CTL on residual viremia during ART must be minor compared to virological factors such as pre-therapy VL (Palmer et al., 2008), and the duration and composition of ART (Bonora et al., 2009).

## EFFECTOR FUNCTION OF HIV-SPECIFIC CTL DURING ART

**FIGURE 1:**
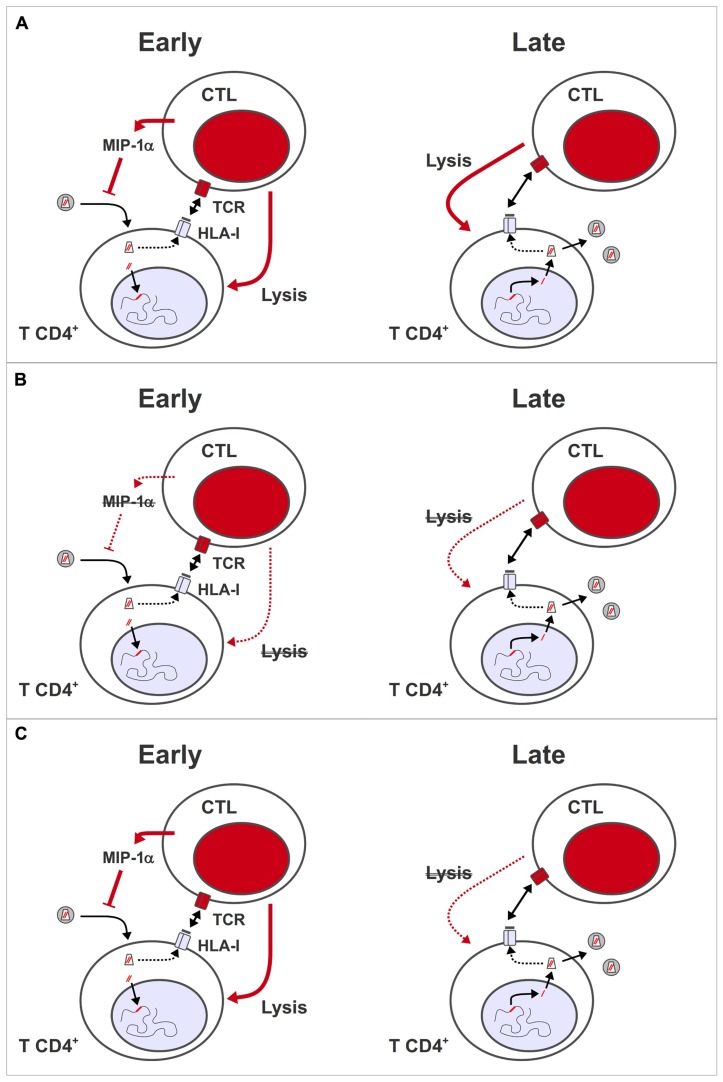
**Antiviral activity of HIV-specific CTL during early and late phases of virus replication**. **(A)** During the pre-integration stages of HIV replication, virus-specific CD8^+^ T lymphocytes can inhibit viral replication through the release of soluble mediators, such as MIP-1α, which blocks virus entry, and/or by lysing infected cells presenting peptides derived from incoming capsid antigens. During the post-integration stages, virus-specific CD8^+^ T lymphocytes lyse cells expressing viral genes from integrated provirus, thus reducing the number of progeny virions that are released per infected cell. **(B)** *Model 1*: HIV-specific CTL are functionally impaired, and/or present in insufficient numbers during ART, so HIV-infected cells cannot be eliminated effectively. This deficiency may be corrected by therapeutic vaccination. **(C)** *Model 2*: HIV-specific CTL only have an impact *in vivo* during the early stages of virus replication, so have no effect on the release of virus from reactivated latent provirus. Even if CTL remain functionally competent or are boosted through therapeutic vaccination during ART, they have no impact on viral reservoirs.

Finally, quality as well as quantity appears to influence the impact of HIV-specific CTL on the virus. This concept was highlighted by studies of HIV controllers, who maintain VL at a level of <500 copies/mL in the absence of ART (Grabar et al., 2009). HIV-specific CTL from controllers have a higher proportion of multi-functional effector cells, capable of secreting tumor necrosis factor-alpha (TNF-α) and interleukin-2 (IL-2) in addition to IFN-γ and macrophage inflammatory protein 1 beta (MIP-1β), and show greater proliferation after stimulation with antigen (Betts et al., 2006; Migueles et al., 2008; Pereyra et al., 2008; Almeida et al., 2009) compared to HIV-specific CTL from patients with chronic progressive infection. In addition, CD8^+^ T lymphocytes from most HIV controllers show rapid *de novo* perforin expression (Hersperger et al., 2010), and can suppress HIV replication in an *ex vivo* assay (Sáez-Cirión et al., 2009). Control of HIV replication in this assay was dependent on cell–cell contact, and did not involve soluble inhibitors of HIV replication (Sáez-Cirión et al., 2007), suggesting that highly efficient lysis of infected target cells is what enables CD8^+^ T cells from HIV controllers to suppress HIV replication *in vivo* (**Figure 1A**).

If the ability to suppress virus replication in an *ex vivo* assay is a valid measure of *in vivo* antiviral efficacy, to what extent do HIV-specific CTL recover this capacity during ART? Two longitudinal studies found that the functional “exhaustion” of HIV-specific CTL (Day et al., 2006; Trautmann et al., 2006; Jones et al., 2008) observed in viremic patients is reversed during ART (Rehr et al., 2008; Streeck et al., 2008). However, several studies found that the functional capacities of HIV-specific CTL from patients on fully suppressive ART remain inferior to those observed in HIV controllers. In particular, HIV-specific CTL from controllers maintained a greater proliferative capacity, and displayed more efficient suppression of HIV replication than CTL from patients on ART (Migueles et al., 2008, 2009; Sáez-Cirión et al., 2009; Shan et al., 2012).

Antiretroviral therapy therefore appears to restore many effector functions in HIV-specific CTL, but at the same time, the number of circulating HIV-specific cells decreases, and their ability to lyse infected cells remains inferior to that seen in HIV controllers.

## CONCLUSION

During partially effective ART, in patients with active HIV replication and VL > 1000 copies/mL, the accumulation of CTL escape mutations in virus sequences, and the inverse correlation between Gag-specific CTL and VL both indicate that HIV-specific CTL continue to have an impact on HIV replication. CTL may therefore play a role in limiting the replication of drug-resistant virus during virological failure of ART. In contrast, there is little evidence that CTL continue to exercise an antiviral effector function in patients on fully suppressive ART.

With respect to HIV eradication strategies based on pharmacological reactivation of latent proviruses, the available data suggests that the HIV-specific CTL present in patients on fully suppressive ART will not be able to eliminate HIV-infected CD4^+^ T cells effectively. If this is entirely due to a degree of functional impairment and a quantitative deficit in CTL during ART (**Figure 1B**), then restimulating HIV-specific CTL by therapeutic vaccination may improve the prospects for eradicating latent reservoirs. However, observations in the macaque simian immunodeficiency virus (SIV) model suggest an alternative explanation why HIV-specific CTL do not have an antiviral effector function during ART. By depleting CD8^+^ T cells in SIV-infected animals prior to the initiation of ART, then analyzing the kinetics of VL reduction, two groups showed that both the virological response to ART, and the *in vivo* half-life of productively infected cells were independent of the presence of SIV-specific CTL (Klatt et al., 2010; Wong et al., 2010). These results indicate that *in vivo*, SIV-specific CTL only have an impact on the early stages of the viral replication cycle (**Figure 1C**), either through release of soluble inhibitors of HIV replication (such as the CCR5 ligand, MIP-1α), or by lysis of infected cells before the expression of viral genes, due to the presentation of peptides from incoming virus capsids (Sacha et al., 2007; Payne et al., 2010). If a similar situation pertains in HIV infection, then therapeutic vaccination will not have an impact on HIV reservoirs – even in conjunction with interventions that reactivate latent provirus – because infected cells with a reactivated provirus only go through the later stages of a HIV replication cycle. In this case, immunotoxins that target Env on the surface of HIV-infected cells (Brooks et al., 2003; Berger and Pastan, 2010) may be a more promising approach for HIV eradication.

## Conflict of Interest Statement

The author declares that the research was conducted in the absence of any commercial or financial relationships that could be construed as a potential conflict of interest.
